# Effects of Visual Occlusion on Lower Extremity Biomechanics during a Low-Intensity Single-Leg Landing

**DOI:** 10.5114/jhk/190681

**Published:** 2024-12-19

**Authors:** Satoshi Imai, Kengo Harato, Yutaro Morishige, Takeo Nagura, Hideo Matsumoto, Kimitaka Hase

**Affiliations:** 1 Department of Rehabilitation, Keio University Hospital, Tokyo, Japan.; 2 Department of Physical Medicine and Rehabilitation, Kansai Medical University, Osaka, Japan.; 3 Department of Orthopaedic Surgery, School of Medicine, Keio University, Tokyo, Japan.; 4 Japan Sports Medicine Foundation, Tokyo, Japan.

**Keywords:** visual information, motor control, kinematics, landing movement, anterior cruciate ligament

## Abstract

Visual information is crucial for motor control during a jump-landing, allowing for anticipation of landing timing and prediction of the impact. However, the effects of visual occlusion on lower extremity biomechanics are not well understood. To investigate this, we studied the impact of visual occlusion on motor control during a low-intensity single-leg landing. Seventeen female college students participated in the controlled laboratory investigation. They performed low-intensity repetitive vertical hopping on a single leg under eyes-open (EO) and eyes-closed (EC) conditions. Main outcome measurements were taken, including jump height, ground reaction forces, joint angles, and joint moments, using a motion capture system. The significant effects of visual occlusion were as follows: 1) a decrease in the hip flexion angle at ground contact (p = 0.02), 2) an increase in Fx (medio-lateral ground reaction force), knee valgus, and internal rotation angles in the early phase within 80 ms after ground contact (p < 0.05), and 3) an increase in Fz (vertical ground reaction force) and a reduction in hip and knee flexion angles at peak Fz (p < 0.05). The amount of angular change at the ankle joint correlated with the hip and knee joints only under the EC condition (p < 0.05). These changes indicate modifications in landing strategy for safety and/or deficiencies in control for an efficient and accurate landing. In conclusion, visual information contributes to safe and accurate motor control during low-intensity landing movements.

## Introduction

Vision, along with vestibular and somatosensory systems, plays a crucial role in motor control during landing movements. A landing from a jump involves rapid motion, high impact forces, and significant joint moments. In addition to motor functions such as muscle strength and balance, pre-landing preparation is essential for ensuring safety, efficiency, and precise control of this motion.

The visual system provides information to the central nervous system (CNS) about the jump height and floor conditions. The CNS uses this visual information to anticipate the timing of ground contact, predict the impact forces upon the landing, and modulate muscle activity (muscle stiffness) 50–100 ms prior to ground contact (Duncan and Mcdonagh, 2000; Horita et al., 1996; Prieske et al., 2013; Taube et al., 2012a). For instance, muscle activity before and after the landing is greater when jumping from a higher platform compared to a lower one (Arampatzis et al., 2003; Santello and Mcdonagh, 1998). Studies have shown that when visual information is occluded during drop-landings from a high platform, muscle activity in the lower extremities increases, resulting in higher joint stiffness during the landing (Chu et al., 2012; Santello et al., 2001). These findings indicate the crucial role of vision in regulating muscle and joint stiffness during landing movements and highlight the impact of visual occlusion on movement efficiency and lower extremity kinetics.

However, there is limited research on the influence of visual occlusion on movement accuracy and lower extremity kinematics during the landing (Louw et al., 2015). Especially, the influence on a single-leg landing, which has a high risk for sports injuries such as the anterior cruciate ligament (ACL) rupture and the Achilles tendon rupture, remains unknown. Additionally, although ACL injuries occur during three-dimensional complex motion (Koga et al., 2010; Krosshaug et al., 2007), the influence of visual occlusion on frontal and horizontal motion also remains unclear. Therefore, the purpose of this study was to investigate the impact of visual occlusion on three-dimensional kinematics during a single-leg landing. In addition to Chu et al.’s (2012) and Santello et al.’s (2001) reports on visual occlusion in a double-leg landing, there are reports highlighting that a single-leg landing has higher ground reaction force and knee valgus than a double-leg landing (Pappas et al., 2007; Yeow et al., 2011). Based on these reasons, we hypothesized that visual occlusion would affect lower extremity biomechanics with injury risk during a low-intensity single-leg landing.

## Methods

### Participants

Seventeen female college students participated in this study (mean age 19.6 ± 1.5 years, body height 1.63 ± 0.05 m, body mass 56.9 ± 4.8 kg). They were members of college sports teams (13 basketball players and four soccer players) and practiced their sport for at least three hours a day, five days a week. None of them had a history of severe injury in the trunk or lower extremities. Since landing biomechanics differ between females and males (Boguszewski et al., 2015; Cronström et al., 2016a; Ford et al., 2010a), only female participants were recruited for this study. The study received approval from the Institutional Review Board of the Keio University (protocol code: 20080054; approval date: 01 April 2016), and all athletes provided informed consent prior to participation.

### Design and Procedures

**Figure 1:**
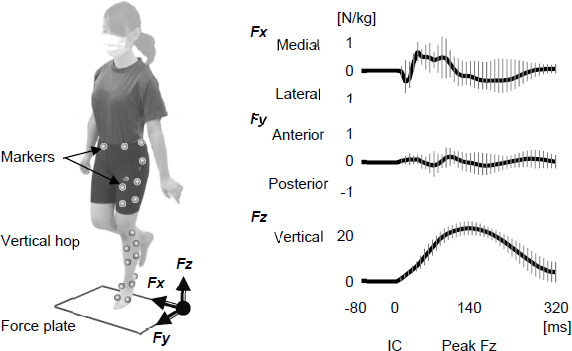
Experimental set-ups and the ground reaction force-time curves in the eyes-open condition. IC: initial contact. Bold lines: means, Vertical thin line: 2 standard deviation

Participants stood in the center of a force plate and performed five consecutive vertical single-leg hops on the non-dominant leg (Figure 1). The dominant leg was determined by asking which leg they preferred to kick a ball, based on a previous study on ACL injury (Brophy et al., 2010; Ruedl et al., 2012). They were verbally instructed to hop with less than half effort and without anxiety. After two practice sets, participants performed single-leg hops under two conditions: eyes open (EO) and eyes closed (EC). Participants first completed the EO condition, followed by the EC condition. They were verbally instructed to maintain the same intensity level during hopping under both conditions.

The single-leg hop tests were recorded using a motion analysis system comprising eight cameras (120 frames/s; Oqus, Qualisys, Sweden), a force plate (frequency 600 Hz; AM6110, Bertec, Columbus, OH, USA), and 40 retroreflective markers (14 mm in diameter) (Figure 1). Marker placement and motion evaluation algorithms followed previous research on single-leg motion analysis (Harato et al., 2019; Whatman et al., 2013). An anatomical model was created by digitizing standard bony landmarks, including bilateral anterior and posterior superior iliac spines, bilateral iliac crests, the greater trochanter, the lateral and medial femoral epicondyle, the lateral and medial malleoli, the posterior heel, the medial cuneiform, the great toe, and heads of the 5^th^ metatarsal. Four additional tracking markers were placed on the frontal aspects of each thigh and shank. All markers were directly attached to the skin using double-sided adhesive tapes and positioned by an experienced orthopedic-sports physiotherapist.

The Qualisys Track Manager software (version 2.7) recorded the marker locations and motions. First, a reference standing posture was established for motion analysis, and segments and joint centers were identified using biomechanical analysis software (Visual 3D, C-motion Company, Rockville, MD, USA). Lower limb segments were modeled as frusta of cones, forming a 6-degree-of-freedom, rigid link biomechanical model. Joints were defined as the meeting points between the distal end of one segment and the proximal end of another segment (Whatman et al., 2013). Three-dimensional kinematics (joint angle, °) and kinetics (joint moment, Nm/kg) of the lower extremities during hopping were calculated using Visual 3D. For ankle kinematics, sagittal plane motion was expressed as plantarflexion-dorsiflexion, frontal plane motion as inversion-eversion, and horizontal plane motion as internal rotation-external rotation, following the recommendations of the International Society of Biomechanics (Wu et al., 2002). Joint moments were calculated using the inverse dynamics model and described as “internal moment”.

### Main Outcome Measures

Jump height during hopping was measured based on the location of the anterior superior iliac spines on the hopping side. Regarding the ground reaction force, maximum values of mediolateral (Fx), anteroposterior (Fy), and vertical (Fz) forces, as well as the time from initial contact to each maximum value, were measured by developing force-time curves (Figure 1). Additionally, the force impulse, defined as the integral of Fz from initial contact to take-off, was computed. The maximum values and impulses were normalized by jump height, dividing each value by jump height.

Joint angles were analyzed by developing angle-time curves for the hip, knee, and ankle joints from 80 ms before initial contact to take-off. The angle at initial contact, at peak (maximum angle), at maximum ground reaction forces, and the time from initial contact to the maximum knee joint angles were computed. The amount of angular change in the sagittal, frontal, and horizontal planes was also calculated, as joint motion may alter across the neutral (0 degrees) angle in each plane of motion. Regarding joint moments, maximum values were calculated. Individual values were averaged over three out of five consecutive hops, excluding the first and last hops.

### Statistical Analysis

All data were presented as mean ± standard deviation. To examine the effects of visual occlusion, differences between the EO and EC conditions were analyzed using the Wilcoxon's signed-rank test. The Spearman's correlation test was used to evaluate the relationship among hip, knee, and ankle joint kinematics. The level of statistical significance was set at 0.05. All analyses were conducted using SPSS (ver. 24, IBM Corporation, Armonk, NY, USA).

## Results

### Jump Heights and Ground Reaction Forces

Table 1 provides data concerning jump height and ground reaction forces. Jump height was significantly lower under the EC compared to the EO condition. Furthermore, the EC condition exhibited significantly increased values for maximum Fx, Fz, and force impulse compared to the EO condition. However, there were no significant differences observed in the times from initial contact to the maximum Fx, Fy, and Fz between the EC and EO conditions.

**Table 1:** Jumping height and ground reaction force.

Condition / *p* value	EO	EC	*p* value
**Jumping heights (cm)**	9.9 ± 3.7	9.1 ± 2.8	0.004^*^
**Maximum ground reaction forces (N/kg)**			
Fx	1.6 ± 0.4	2.0 ± 1.0	0.049^*^
Fy	0.8 ± 0.2	0.8 ± 0.2	0.356
Fz	25.9 ± 2.6	26.7 ± 2.4	0.010^*^
**Maximum ground reaction forces (N/kg/jumping height)**			
Fx	0.17 ± 0.01	0.23 ± 0.10	0.007*
Fy	0.08 ± 0.04	0.10 ± 0.05	0.165
Fz	2.78 ± 0.58	3.12 ± 0.75	<0.001*
**Impulses (N/kg)**			
	597 ± 68	627 ± 80	<0.001*
**Impulses (N/kg/jumping height)**			
	70.1 ± 19.8	73.7 ± 22.0	<0.001*
**Times from IC to peak ground reaction forces (ms)**			
Fx	79 ± 25	77 ± 27	0.633
Fy	164 ± 89	136 ± 94	0.326
Fz	146 ± 28	149 ± 28	0.938

EO: eyes-open, EC: eyes-closed, Fx: medial-lateral ground reaction force, Fy: anterior-posterior force, Fz: vertical force, Asterisks (*): Significant difference between EO and EC

### Lower Extremity Kinematics and Kinetics

**Figure 2:**
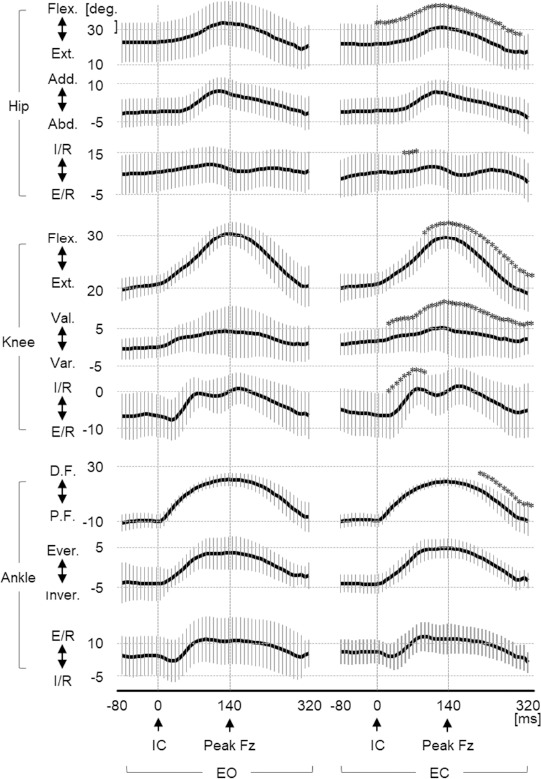
Joint angle-time curves. EO: eyes-open, EC: eyes-closed, IC: initial contact. Bold lines: means, Vertical thin lines: 2 standard deviation, Asterisks (*): significant difference between EO and EC

Figure 2 illustrates the joint angle-time curves, and Table 2 presents the angles at initial contact and at peak of the hip, knee, and ankle joints. At the initial contact, only the hip flexion angle showed a significant change, decreasing under the EC condition compared to the EO condition. Compared to the maximum angle values, the hip flexion and knee flexion angles were significantly decreased, while the knee valgus and knee internal rotation angles were significantly increased under the EC compared to the EO condition. The time from initial contact to the maximum knee flexion angle was significantly shorter under the EC (152 ± 26 ms) compared to the EO condition (162 ± 32 ms).

**Table 2:** Joint angles at initial contact and maximum joint angles.

Timing	At Initial contact			Maximum value		
	EO	EC	*p* value	EO	EC	*p* value
**Joint angle (deg.)**						
**Hip joint**						
Flexion	23.4 ± 10.3	22.3 ± 10.6	0.022*	34.3 ± 13.2	31.7 ± 12.0	0.001*
Adduction	−1.4 ± 5.5	−1.1 ± 5.3	0.523	7.2 ± 5.3	7.0 ± 5.1	0.831
I/R	6.2 ± 9.2	5.7 ± 9.0	0.906	12.3 ± 9.3	11.4 ± 8.7	0.163
**Knee joint**						
Flexion	22.2 ± 5.5	22.2 ± 6.4	0.943	52.8 ± 6.9	50.9 ± 7.4	0.028*
Valgus	0.2 ± 3.2	0.6 ± 3.6	0.246	3.4 ± 4.1	4.1 ± 4.5	0.039*
I/R	−7.2 ± 6.0	−6.7 ± 6.5	0.523	3.0 ± 4.7	4.3 ± 5.4	0.013*
**Ankle joint**						
D/F	−12.3 ± 6.6	−11.7 ± 6.1	0.381	22.5 ± 7.9	19.7 ± 5.9	0.062
Eversion	5.6 ± 5.9	5.7 ± 3.9	0.309	9.5 ± 5.3	9.2 ± 4.0	0.356
E/R	3.4 ± 8.6	5.2 ± 5.2	0.435	13.5 ± 10.4	14.0 ± 6.6	0.981

EO: eyes-open, EC: eyes-closed, IR: internal rotation, D/F: dorsi-flexion, ER: external rotation, Asterisks (*): Significant difference between EO and EC

Regarding the knee joint angles at the maximum ground reaction forces, the flexion angle at the maximum Fx was significantly decreased, and the valgus angle at the maximum Fx was significantly increased under the EC compared to the EO condition. The flexion and valgus angles at the maximum Fz significantly decreased under the EC compared to the EO condition.

The amount of angular change in the sagittal plane showed significant correlations among the hip, knee, and ankle joints under both the EC and EO conditions (Table 3). Under the EC condition, the amount of angular change in the horizontal plane also showed significant correlations among each joint.

**Table 3:** Correlation coefficient of the amount of angular changes among hip, knee and ankle joints.

		EO		EC	
**Sagittal**		**Hip**	**Knee**	**Hip**	**Knee**
**Knee**	r	0.853	-	0.828	-
	*p*	<0.001*	-	<0.001*	-
**Ankle**	r	0.571	0.831	0.542	0.831
	*p*	0.017*	<0.001*	0.025*	<0.001*
**Frontal**		**Hip**	**Knee**	**Hip**	**Knee**
**Knee**	r	0.047	-	−0.184	-
	*p*	0.859	-	0.480	-
**Ankle**	r	0.385	0.441	−0.012	0.429
	*p*	0.127	0.076	0.963	0.086
**Horizontal**		**Hip**	**Knee**	**Hip**	**Knee**
**Knee**	r	0.252	-	0.569	-
	*p*	0.328	-	0.017*	-
**Ankle**	r	−0.024	0.387	0.499	0.620
	*p*	0.972	0.125	0.041*	0.008*

EO: eyes-open, EC: eyes-closed, r: correlation coefficient, Asterisks (*): Significant correlation between the movement of those two joints

Regarding the maximum knee joint moments, the extension moment was 2.71 ± 0.64 Nm/kg under the EO and 2.86 ± 0.55 Nm/kg under the EC condition, and the valgus moment was 1.19 ± 0.44 Nm/kg under the EO and 1.20 ± 0.45 Nm/kg under the EC condition. No significant differences were found between the EO and EC conditions.

## Discussion

**Figure 3:**
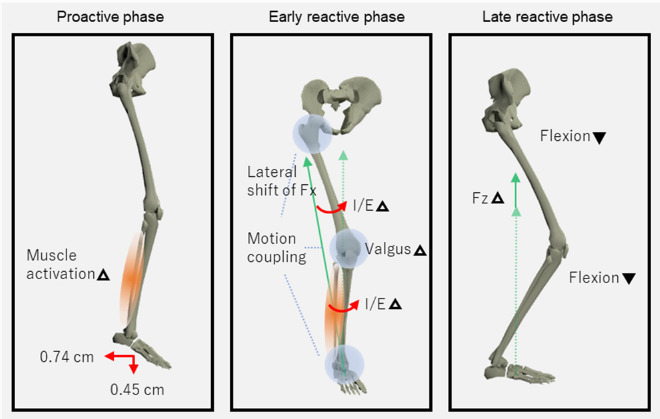
Effects of occluded vision on landing biomechanics. △, ▼ : increased and decreased values in the eyes-closed condition compared with the eyes-open condition, I/E: internal rotation

In this study, we observed several significant effects of visual occlusion on lower extremity biomechanics (Figure 3). First, visual occlusion led to a decrease in the hip flexion angle at initial contact. Second, it resulted in an increase in maximum Fx (horizontal force), the knee valgus angle, and the knee internal rotation angle during the period from initial contact to the maximum Fz (vertical force). Third, visual occlusion was associated with an increase in the maximum Fz and a decrease in hip and knee flexion angles at the maximum Fz. Motor control during the jump-landing and hopping can be divided into different phases, including before ground contact, early reactive phase (40–80 ms after ground contact), and late reactive phase (Komi, 2003). These phases are regulated by a combination of anticipatory muscle contractions, spinal reflexes, and long latency responses (Komi, 2003; Leukel et al., 2012). In this discussion, we explore the motor control strategies employed during the proactive, early reactive, and late reactive phases of hopping motion and discuss the impact of visual occlusion on low-intensity movement.

### Motor Control in the Proactive Phase

Motor control in the proactive phase involves kinematic changes at initial contact, particularly in the hip joint, where a significant decrease in the flexion angle was observed with visual occlusion (EC) compared to the control condition (EO) (Table 2). The initial contact location of the toe was also displaced. Specifically, it was displaced approximately 0.45 cm downward and 0.74 cm backward compared to the condition where visual information was available (Figure 3). These measurements were obtained by applying joint angle modifications in the sagittal plane, taking into account the average values of thigh length, shank length, and foot length specific to the female college students participating in the study (Inoue, 1999).

Participants seemed to extend their hip joint, resulting in a lower and closer initial contact location to the floor and the center of gravity line in the absence of visual information. The timing and intensity of the impact during the landing in hopping motion can be predicted since it is a result of a motor command. Therefore, joint kinematics the before landing is adjusted to optimal angles through kinematic commands from the CNS (Central nervous system) with reference to an internal model (Avela et al., 1996; Márquez et al., 2014; Taube et al., 2012b; Zuur et al., 2010).

An excessive increase in lower extremity flexion at initial contact leads to insufficient time and force for adequate weight support, while a reduced flexion angle increases strain on the joint and the risk of injury. Effective shock absorption requires an appropriate flexion angle at initial contact and sufficient joint mobility afterward. Kinematic modification at initial contact was only observed in the hip joint, not in the knee or ankle joints (Table 2). The posterior and downward displacement of the initial contact location resulting from hip extension helps reduce joint moments in the lower extremity. On the other hand, maintaining knee and ankle joint angles facilitates joint mobility and shock absorption after initial contact. Previous research has reported that the ankle and knee joints play a more significant role in hopping motion compared to the hip joint (Hobara et al., 2009, 2011). Therefore, we may conclude that participants made minor adjustments to their contact location through hip motion during the proactive phase without altering the kinematics of the knee and ankle joints, which is crucial for hopping performance.

### Motor Control in the Early Reactive Phase

Motor control in the early reactive phase involves significant kinematic changes in the hip and knee joints, characterized by increased hip and knee internal rotation and knee valgus (Figure 2). In the frontal plane motion, knee joint modifications were not associated with adjustments in the hip or the ankle joint, but rather correlated with the ground reaction force. The peak ground reaction force in the frontal plane (Fx) occurred 40–80 ms after initial contact (Figure 1), and it was significantly higher under the visual occlusion condition compared to the control condition (Table 1). Furthermore, a significant increase in the knee valgus was observed at the maximum Fx. These findings suggest that visual occlusion led to the knee valgus associated with increased Fx during the early reactive phase after initial contact. The increase in Fx indicates a shift in the center of gravity toward the hopping leg side, reducing the lever arm for the gluteus medius muscle, which plays a significant role in stable control on the frontal motion (Mclelsh and Charnley, 1970). Based on this, we can conclude that participants shifted their center of gravity toward the hopping leg side to steadily manage their center of gravity on the base of support in the absence of visual feedback, resulting in increased Fx and knee valgus in the early reactive phase (Figure 3).

In terms of horizontal motion, there was a significant increase in hip and knee internal rotation angles, while ankle joint rotation showed no significant change (Figure 2). However, the amount of angular change in ankle rotation was significantly correlated with the changes in the knee and hip joint rotation, under the condition of visual occlusion (Table 3). Athletes with chronic ankle instability have been reported to increase ankle and knee joint stiffness, along with coupling between the knee and hip joints, to stabilize their landings (Li et al., 2021). Visual occlusion may have caused a similar strategy of increased ankle-knee-hip coupling.

In this study, despite the immediate increase in Fx following initial contact under the visual occlusion condition, the foot (ankle joint), which is the effector to the floor, exhibited the same motion as under the control condition. The increase in Fx indicates a shift in the center of gravity toward the hopping leg side and a displacement of the center of foot pressure toward the medial side. These alignments result in internal rotation of the shank, the thigh, and the knee joint (Khamis and Yizhar, 2007; Motooka et al., 2012). We may thus conclude that the effect of visual occlusion on horizontal plane motion began with the change in Fx and extended upward through the coupling motion of the foot, knee, and hip joints (Figure 3).

### Motor Control in the Late Reactive Phase

In the late reactive phase, the visual occlusion condition resulted in an increase in peak Fz value and force impulse, despite a reduction in jump height (Table 1). These outcomes were associated with the control of knee flexion. When individuals of the same body weight land at the same velocity, the Fz and force impulse decrease as the time for shock absorption and knee flexion increases. In this study, participants under the visual occlusion condition exhibited a decrease in the time to reach maximum knee flexion and a decrease in the maximum flexion angle. The increase in Fz and the decrease in knee flexion due to visual occlusion have been observed in previous research. Santello et al. (2001) and Chu et al. (2012) reported this effect and suggested that high preparatory contraction during the proactive phase led to a “stiff-landing” with reduced knee flexion and increased impact. Additionally, hopping is a cyclic motion involving stretch-shortening, and efficient energy transfer from eccentric to concentric motion is critical (Komi, 2003). It has been reported that increasing hopping intensity, such as frequency and jump height, results in increased muscle activity in the thigh and the shank, as well as increased joint stiffness (Hobara et al., 2007; Kuitunen et al., 2011). In this study, participants adjusted their jump height to ensure safe landings under the visual occlusion condition. However, they also increased their stretch-shortening cycle activity to maintain hopping performance, resulting in landings with less knee flexion and higher Fz.

Regarding frontal motion, we also found a significant increase in the knee joint valgus following the early reactive phase. In the lateral shift of the center of gravity, an increase in Fz leads to an increase in the knee valgus. Furthermore, a decrease in the flexion angle in the sagittal plane leads to an increase in passive strain in the frontal plane (Pollard et al., 2010). We consider that these factors are associated with an increase in the knee valgus during the late reactive phase.

### Clinical Significance

The findings of this research hold clinical significance, providing insights into biomechanical adaptations and implications for a safe landing when visual information is occluded. Although female college athletes were capable to perform hopping even without visual feedback, detailed analysis revealed necessary adjustments in lower extremity kinematics and kinetics. Adaptations for a safe landing under the visual occlusion condition included reducing jump height, modifying the initial contact point, and shifting the center of gravity toward the hopping leg. These adaptations are considered beneficial for ensuring stability and minimizing the risk of injury.

On the other hand, certain changes observed during visual occlusion, such as increased Fz, decreased hip and knee flexion, and an increased knee valgus, are not recommended for a safe landing as they pose a risk of knee ACL injury (Kiapour et al., 2016; Koga et al., 2010; Shin et al., 2011; Withrow et al., 2006). The clinical significance of this study lies in demonstrating the importance of visual information in athletes' sports movements and its relation to the risk of injury. Thus, while certain modifications aid stabilization for a landing and performance maintenance, others pose a risk for ACL injury. Although changes in Fz, knee valgus, internal rotation, and flexion are small, their accumulation has been suggested to lead to a critical incident for ACL injury (Kiapour et al., 2016; Koga et al., 2010; Shin et al., 2011; Withrow et al., 2006). Furthermore, it is noteworthy that the negative effects of visual occlusion were observed not only in high-intensity tasks, as previously shown in the study by Santello et al. (2001), but also in the low-intensity tasks examined in the present study. These results suggest that visual information plays an important role in promoting safe, efficient, and accurate landings, even during low-intensity movement, supporting the hypothesis that visual occlusion induces a high-risk landing for knee ACL injury. However, further research is needed to link this to ACL injury risk, as the hopping task in this study differed from the actual intensity of injury, and ACL-injured patients and reconstructed patients show different three-dimensional kinematics than normal subjects (Lepley and Kuenze, 2018; Trigsted et al., 2017; Warathanagasame et al., 2023).

Overall, this study emphasizes the significance of vision in motor control and landing performance, underscoring the need to consider visual feedback and motor response when assessing and training individuals in tasks involving landing and lower extremity movements.

### Limitations

In this research, it is important to acknowledge several limitations regarding the hopping task and the outcome measures. Low-intensity hopping was selected as the experimental task to investigate lower extremity biomechanics during a single-leg landing. One advantage of this task is that it reduces psychological anxiety under the EC condition. However, caution is needed when interpreting the clinical relevance of the findings, as the intensity and movement pattern differ from other tasks such as walking and jump-landings. Additionally, it should be noted that in this task, the timing of the landing may not have been entirely masked by the residual memory of the movement.

Regarding the limitations of the outcome measures, the study did not include measurements of electromyography, trunk movement, and the center of gravity. Consequently, it was not possible to directly assess muscle activity before initial contact and trunk stability associated with knee injury (Vermeulen et al., 2023).

Furthermore, the sample size was small. To perform the Wilcoxon’s signed-rank test with an effect size of 0.8, alpha of 0.05, and power of 0.95, the required sample size was 24.

Further research and analysis are required for a comprehensive discussion. Despite these limitations, the study provides insights into the effects of visual occlusion on lower extremity biomechanics during single-leg, low-intensity movement.

## Conclusions

Visual occlusion during low-intensity hopping revealed significant changes in lower extremity biomechanics such as Fz and joint kinematics, highlighting the critical role of vision in optimizing movement accuracy and safety. These findings emphasize the importance of visual feedback in motor control and may have implications for injury prevention and rehabilitation strategies.
